# Violence Perpetration in Early Adolescence: A Study of Four Urban Communities Worldwide

**DOI:** 10.1016/j.jadohealth.2022.06.011

**Published:** 2022-11

**Authors:** Sam Beckwith, Chaohua Lou, Kristien Michielsen, Eric Mafuta, Siswanto Agus Wilopo, Robert Wm Blum

**Affiliations:** a Department of Population, Family, and Reproductive Health, Johns Hopkins Bloomberg School of Public Health, Baltimore, Maryland; b Department of Epidemiology & Social Science, NHC Key Lab. of Reproductive Regulation (Shanghai Institute for Biomedical and Pharmaceutical Technologies), Fudan University, Shanghai, China; c International Centre for Reproductive Health, Ghent University, Ghent, Belgium; d Health Systems Policy and Management Department, University of Kinshasa School of Public Health, Kinshasa, Democratic Republic of the Congo; e Department of Biostatistics, Epidemiology and Population Health and Center for Reproductive Health, Faculty of Medicine, Public Health and Nursing, Universitas Gadjah Mada, Yogyakarta, Indonesia

**Keywords:** Adolescent, Early adolescence, Peer-violence perpetration, Bullying, Adverse childhood experiences, Gender norms

## Abstract

**Purpose:**

Violence perpetration is common among adolescents worldwide but existing research largely focuses on boys, older adolescents, and partner violence. Our study sought to identify individual, family, and neighborhood/peer factors associated with violence perpetration in a multinational sample of male and female young adolescents.

**Methods:**

We used cross-sectional data from 5,762 adolescents in four sites in the Global Early Adolescent Study: Flanders, Belgium; Kinshasa, Democratic Republic of the Congo; Shanghai, China; and Semarang, Indonesia. Adolescents resided in high-poverty urban areas and were aged 10 to 14 years. Logistic regression examined pooled and stratified associations between independent variables with peer violence perpetration in the past six months. Factors included media viewing habits, gender norms, victimization, agency/empowerment, adversity, depression, familial relationships, neighborhood cohesion, and peer behaviors.

**Results:**

Restricted-model analyses found increased odds of violence perpetration associated with high media consumption, pornography viewing, violence or bullying victimization, having drank alcohol, depressive symptoms, adverse childhood experiences, greater behavioral control, greater decision-making, feeling unsafe in the neighborhood/school, peer alcohol/tobacco use, and witnessing peers start a fight. Decreased odds of violence perpetration were associated with more egalitarian views on two gender norms scales, closer parental relationships, neighbors looking out for one another, and greater availability of adult help.

**Discussion:**

Among young adolescents, increased odds of violence perpetration were related to a perceived lack of safety and risky peer behaviors. Parental and neighborhood connections were often associated with decreased perpetration. Further research examining the interplay of such factors among young adolescents is needed to inform effective intervention and policy.

**Implications and Contribution**

This study identifies numerous individual, family, neighborhood, and peer factors associated with the risk of peer-violence perpetration among young adolescents in several countries. It suggests several factors that are shared across sexes and regions, indicating potential opportunities for prevention and intervention in adolescents, families, and peer groups.

There is extensive evidence that youth violence victimization and perpetration are major health issues globally, with profound short-term and life-long sequalae, including poorer mental health and a greater risk of offending [1,2]. Worldwide, one in three adolescents experiences physical violence or bullying, with rates for bullying ranging between 22.8% in Central America and 48.2% in sub-Saharan Africa; the prevalence of physical fighting similarly ranges from 25.6% in Central America to 46.3% in North Africa [3]. Data from the 2019 Youth Risk Behavior Survey found that 8.7% of American high-school students avoided school at least once in the past 30 days because of fear of violence, 7.3% were forced to have sex against their will, and 8.2% experienced dating violence [4].

There is also substantial evidence that many young people who are victims of violence are also perpetrators. In Pakistan, Karmaliani et al. [5] found that large percentages of sixth graders who were victims of violence were also engaged in perpetration (46.4% of girls vs. 72.6% of boys). As Logan-Greene et al. [6] have noted, interpersonal violence perpetration among adolescents is significantly higher among those who have experienced maltreatment or witnessed violence. Using the Youth Risk Behavior Survey data with more than 136,000 adolescents in Minnesota, Duke et al. reported strong associations between adolescent violence perpetration and experienced physical or sexual abuse or having witnessed abuse in the household [7]. Similar relationships have been reported in China [8].

In the United States, Zych et al. [9] have seen a strong relationship between school bullying and dating violence among adolescents. Likewise, in a study of 10–12-year-olds in Sweden, Johansson and Englund found a positive relationship between cyberbullying and physical aggression and relational bullying [10].

Given the prevalence of youth violence, and the consequences of perpetration and victimization to both the victim and the perpetrator, developing effective prevention programs requires a better understanding of the drivers of violence perpetration at the time when interpersonal violence first escalates—during early adolescence [11]. While there is a growing understanding of such factors in low-income and middle-income countries [12], much of the existing research comprises studies in a single country or economic context and few studies focus on young adolescents.

The present study explores violence perpetration in four communities: Flanders, Belgium; Shanghai, China; Kinshasa, Democratic Republic of the Congo (DRC); and Semarang, Indonesia. In this hypothesis-generating study, we focus on young adolescents in each setting (aged 10–14 years) with the aim of identifying common and unique factors, spanning multiple ecological levels [13], that are associated with violence perpetration by geography and respondent sex.

## Methods

### Sample

Data for the present study are derived from the Global Early Adolescent Study, a multisite study examining gender norms and their consequences on health and social functioning across adolescence within the context of urban low-resource settings (https://www.geastudy.org). The present analyses were based on data from four Global Early Adolescent Study (GEAS) sites with available violence data and representing several regions of the world (East Asia, Southeast Asia, Central Africa, and Western Europe). A convenience sample of adolescents in each site was selected using a two-stage sampling procedure consisting of school sampling, followed by the inclusion of students in the selected schools. In all sites, recruitment was based on the following eligibility criteria: aged 10–14 years at enrollment, living in low-income urban centers, and having adolescent assent and parent consent for study participation. Eligibility for the present study was based on these criteria and having completed baseline survey questions related to violence perpetration and respondent's sex.

Altogether, data were available for 5,830 adolescents across all sites. Those with interviews that were assessed negatively on two or more aspects of data quality by interviewers, with one identified as poor response accuracy or compromised understanding of survey questions (n = 8) and those missing more than 15% of survey items (n = 60) were excluded from analyses. The overall sample available for this analysis was 5,762 (2,823 boys and 2,939 girls) with the following distribution by site: 1,985 from Kinshasa, 837 from Flanders, 1,338 from Semarang, and 1,602 participants from Shanghai.

### Measures

Data collection took place between June 2017 and June 2019; the survey was self-administered on tablets using the SurveyCTO platform [14], except in Kinshasa where, due to literacy concerns, computer-assisted in-person interviews were used. The questions were translated into local languages and back-translated into English to assure the meanings were consistent across sites. The survey collected information on a range of topics including young people's sociodemographic characteristics; family, peer, school, and neighborhood environments; physical, mental, and sexual health; and perceptions of gender norms (all measures are available on the GEAS website, https://www.geastudy.org). Each site's survey, except Flanders', included items assessing adverse childhood experiences (ACEs). In Flanders, the Ethical Review Board declined approval for those questions.

### Ethical approval

The GEAS received an ethical approval from the Johns Hopkins Bloomberg School of Public Health Institutional Review Board and from each of the partner institutions of which co-authors are members (details available on request). In addition, the study in Shanghai, China received an Ethical Review Board approval from the World Health Organization.

### Outcome measure

The outcome of interest for the present analysis is peer violence perpetration, operationalized as an affirmative response to one or both of two questions: “During the past six months, have you slapped, hit, or otherwise physically hurt another boy or girl in a way that they did not want?” and “During the past six months, have you bullied or threatened another boy or girl for any reason?” This two-item measure has been applied in existing research with early adolescents [15,16].

### Independent variables

This study sought to investigate potentially intervenable factors reported to be associated with peer violence perpetration that span individual, family/household, and neighborhood/peer levels. Within individual-level factors were three scales related to endorsement of traditional gender norms: sexual double standard, gender stereotypical traits, and gender stereotypical roles [17]. Measures of agency/empowerment [18], depression, and ACEs were also considered. Demographic variables included in the analyses were the age, sex, and site of respondents. A detailed overview of the independent variables is included in Table 1.

**Table 1:** Independent variables, operational definitions, and cross-site variation

Variable	Operational definition	Cross-site variation
Individual-level factors		
High media consumption	Summing hours spent watching TV and on computer/social media per day, dichotomized at site median (1 = above median)	In Flanders, only includes hours spent on computer/social media.
Viewed pornography	Reports ever viewing pornography	Item was not assessed in Kinshasa and Shanghai.
Egalitarian views on sexual double standard	On a six-item scale capturing endorsement of a sexual double standard, scores at least one standard deviation below the site mean (1 = at least 1 SD below site mean)	Cronbach's alpha ranged from 0.74 (Kinshasa) to 0.79 (Flanders).
Egalitarian views on gender stereotypical traits	On a seven-item scale capturing endorsement of gender-stereotypical traits, scores at least on standard deviation below the site mean (1 = at least 1 SD below site mean)	Cronbach's alpha ranged from 0.64 (Kinshasa) to 0.77 (Flanders).
Egalitarian views on gender stereotypical roles	On a four-item scale capturing endorsement of gender-stereotypical roles, scores at least on standard deviation below the site mean (1 = at least 1 SD below site mean)	Scale was not assessed in Flanders. Cronbach's alpha ranged from 0.47 (Kinshasa) to 0.78 (Semarang).
Victim of violence or bullying	Was teased, called names, or physically hurt in past six months	
High academic performance	Performing “better” or “a lot better” than peers academically versus “similar,” “worse,” or “a lot worse”	
Ever drank alcohol	Has ever had alcohol, except for religious purposes.	
Individual mental health and experiences		
Depression symptoms	Collapsed total of six-item index of self-reported depression symptoms: two or three, four to six, versus zero or one symptoms	
Adverse childhood experiences	Collapsed total of 10-item index of self-reported ACEs: one, two or three, four or five, six to 10, versus zero ACEs	Items were not assessed in Flanders.
Agency/empowerment factors		
High youth voice	Average of four ordinal frequency items (being asked for opinion, listened to when sharing an opinion, asked for advice, and listened to if something is wrong), dichotomized at site median (1 = above median)	Cronbach's alpha ranged from 0.64 (Kinshasa) to 0.78 (Shanghai).
High behavioral control	Average of four ordinal frequency items (deciding what to eat, what clothes to wear, what to do in free time, and who to have as friends), dichotomized at site median (1 = above median)	Cronbach's alpha ranged from 0.65 (Semarang) to 0.75 (Flanders and Shanghai).
High decision-making	Average of three ordinal items related to the level of influence in decision (when to leave school, when to marry, and who to marry), dichotomized at site median (1 = above median)	Cronbach's alpha ranged from 0.56 (Flanders) to 0.85 (Semarang).
Family/household-level factors		
Married parents	Parent/primary caretaker is married or cohabiting as if married, versus not	In Kinshasa and Semarang, this variable was assessed using parent/caretaker-reported data. Flanders and Shanghai used adolescent-reported data for this variable.
High parental education	Parent/head of household has more than secondary school versus secondary school or less	In Flanders and Shanghai, this item was operationalized as college/university versus secondary school or less. In Kinshasa and the Indonesian sites, this variable was assessed using parent/caretaker-reported data. Flanders and Shanghai used adolescent-reported data for this variable.
High parental education expectations	At least one parent expects adolescents to complete higher education/university (Kinshasa) or more than a Bachelor's degree (Semarang).	Item was not assessed in Flanders or Shanghai.
At least one parent employed	At least one parent currently works for pay	In Semarang, this variable was assessed using parent/caretaker-reported or adolescent-reported data. In Kinshasa, this variable was assessed using parent/caretaker-reported data only. Flanders and Shanghai used adolescent-reported data for this variable only.
High parental monitoring	Average of three ordinal items of parental monitoring (knows who my friends are by name, knows my grades/how I am doing in school, and usually knows where I am), dichotomized at the site median (1 = above median)	
Comfortable talking to parent/caretaker about worries	Response of “very,” “somewhat,” or “not very” comfortable” versus “not at all comfortable”	
Very close to parent/caretaker	Response of “very close” versus “not at all,” “not very,” or “somewhat” close	
Neighborhood and peer factors		
Neighbors look out for one another	Response of “very true” versus “not,” “not very,” or “somewhat” true to item “People in my neighborhood look out for others”	
Unsafe school/neighborhood	Endorsement of feeling unsafe in neighborhood or school in the past year	In Semarang, item asked about neighborhood (not neighborhood or school)
Adult help is available	Response of “often” versus “sometimes,” “rarely,” or “never” to item “I can ask adults for help when I need it”	
Peers smoke or drink	Response of “none” versus “few,” “most,” or “all” to at least one of items “In general, how many of your friends do you think smoke cigarettes (tobacco)” and “In general, how many of your friends do you think drink alcohol (store-bought or home-brewed)?”	
Peers have started a fight	Reports seeing male or female peers start a physical fight with someone in the past six months.	

Missing data were less than 3% for most variables; however, missing responses exceeded 3% for the following: parent education, academic performance, neighbors looking out for one another, availability of adult help, decision-making, violence or bullying victimization, pornography viewing, parent educational expectations, and both peer-risk behavior variables. Where missingness exceeded 3%, site-stratified multiple imputation was employed [19].

### Analyses

First, we examined differences in violence perpetration by sex, age, and study site using Chi-squared tests of association (Table 2). Concurrently, we examined site-specific and sex-specific levels of the other independent variables testing significance with Chi-squared tests (Table 3). Subsequently, we estimated pooled and site-specific and sex-specific odds ratios of violence perpetration for each of the independent variables of interest. In restricted-model analyses (Table 4), pooled odds ratios were adjusted for age, sex, and site; site-specific and sex-specific odds ratios were adjusted for age. In full-model analyses (Table 5), site-specific logistic regression models were estimated that initially included all independent variables noted in Table 3 plus age and sex. We subsequently removed independent variables with a variance inflation factor (VIF) more than 10 which indicates substantial multicollinearity [20].

**Table 2:** Violence perpetration in past six months by site, sex, and age

	Violence perpetration		*p* value
	No (N = 4,513)	Yes (N = 1,249)	
Sex			< .001
Male	2,092 (74.1)	731 (25.9)	
Female	2,421 (82.4)	518 (17.6)	
Age			< .001
10	282 (68.6)	129 (31.4)	
11	598 (77.7)	172 (22.3)	
12	1,635 (79.4)	424 (20.6)	
13	1,342 (80.5)	326 (19.5)	
14	656 (76.8)	198 (23.2)	
Site			< .001
Flanders, Belgium	666 (79.6)	171 (20.4)	
Kinshasa, DRC	1,322 (66.6)	663 (33.4)	
Shanghai, China	1,477 (92.2)	125 (7.8)	
Semarang, Indonesia	1,048 (78.3)	290 (21.7)

**Table 3:** Independent variables by site and sex

Factor	Total (N = 5,762)	Flanders, Belgium		Shanghai, China		Kinshasa, DRC		Semarang, Indonesia		*p* value
		Male (N = 457)	Female (N = 380)	Male (N = 807)	Female (N = 795)	Male (N = 961)	Female (N = 1,024)	Male (N = 598)	Female (N = 740)	
Individual-level factors										
High media consumption	3,696 (64.6)	277 (61.8)	229 (62.2)	536 (66.5)	570 (72)	676 (70.5)	588 (57.6)	331 (55.7)	489 (66.5)	< .001
Viewed pornography	528 (26.1)	201 (50.9)	43 (12.2)	-	-	-	-	186 (32.2)	98 (14.0)	< .001
Egalitarian views on sexual double standard	967 (17.2)	86 (20.5)	63 (18.6)	170 (21.4)	92 (11.7)	178 (18.6)	100 (9.8)	134 (22.6)	144 (20.0)	< .001
Egalitarian views on gender stereotypical traits	799 (14)	45 (10.7)	93 (25.9)	82 (10.2)	119 (15.0)	132 (13.7)	106 (10.4)	96 (16.1)	126 (17.1)	< .001
Egalitarian views on gender stereotypical roles	600 (12.3)	-	-	59 (7.5)	220 (28.0)	116 (12.1)	115 (11.3)	38 (6.4)	52 (7.1)	< .001
Victim of violence or bullying	2,516 (45.7)	169 (40.6)	136 (40.4)	352 (46.4)	249 (33.2)	483 (50.3)	359 (35.2)	374 (64.7)	394 (57.5)	< .001
High academic performance	2,347 (42)	193 (44.4)	120 (33.6)	354 (44.3)	363 (46.2)	467 (49.8)	466 (46.1)	158 (28.1)	226 (32.4)	< .001
Ever drank alcohol	793 (14.1)	104 (23.6)	78 (21.5)	219 (28.4)	215 (28.3)	100 (10.4)	55 (5.4)	17 (2.9)	5 (0.7)	
Individual mental health and experiences										
Depression: 2–3 symptoms	1,721 (30.0)	82 (18.3)	89 (23.9)	276 (34.2)	292 (36.8)	240 (25.0)	252 (24.6)	199 (33.3)	291 (39.4)	< .001
Depression: 4–6 symptoms	637 (11.1)	23 (5.1)	39 (10.5)	126 (15.6)	165 (20.8)	49 (5.1)	34 (3.3)	84 (14.1)	117 (15.8)	
Adverse childhood experiences: 1 ACE	1,038 (21.6)	-	-	148 (18.8)	163 (20.8)	239 (24.9)	245 (23.9)	103 (18.5)	140 (19.9)	< .001
Adverse childhood experiences: 2–3 ACEs	1,778 (36.9)	-	-	325 (41.2)	282 (36.1)	343 (35.7)	331 (32.4)	201 (36.1)	296 (42.0)	
Adverse childhood experiences: 4–5 ACEs	733 (15.2)	-	-	135 (17.1)	137 (17.5)	130 (13.5)	101 (9.9)	116 (20.8)	114 (16.2)	
Adverse childhood experiences: 6 + ACEs	228 (4.7)	-	-	33 (4.2)	26 (3.3)	44 (4.6)	45 (4.4)	50 (9.0)	30 (4.3)	
Agency/empowerment factors										
High youth voice	2,515 (44.7)	172 (41.1)	213 (57.7)	295 (38.4)	337 (43.6)	481 (50.1)	440 (43.0)	198 (34.3)	379 (51.8)	< .001
High behavioral control	2,467 (43.8)	151 (35.2)	133 (36.7)	346 (44.8)	387 (49.7)	482 (50.2)	483 (47.2)	198 (34.1)	287 (39.6)	< .001
High decision-making	2,278 (46.5)	171 (44.5)	127 (38.7)	315 (45.9)	362 (52.2)	362 (40.2)	481 (47.3)	202 (50.4)	258 (52.7)	< .001
Family/household-level factors										
Married parents	4,143 (74)	324 (75.9)	268 (74.0)	717 (89.5)	680 (86.3)	453 (49.7)	482 (48.2)	550 (93.2)	669 (92.9)	< .001
High parental education	2,433 (46.2)	201 (65.0)	152 (59.6)	395 (53.6)	411 (55.5)	386 (42.4)	434 (44.0)	220 (37.2)	234 (31.9)	< .001
High parental educational expectations	2,278 (71.5)	-	-	851 (93.3)	907 (91.7)	-	-	242 (42.1)	278 (39.2)	< .001
At least one parent employed	5,059 (90.5)	397 (93.9)	324 (92.0)	786 (98.9)	786 (99.4)	725 (78.4)	841 (83.3)	538 (92.1)	662 (92.7)	< .001
High parental monitoring	2,463 (42.9)	146 (32.4)	170 (44.7)	315 (39.4)	420 (53.0)	388 (40.4)	544 (53.1)	178 (29.8)	302 (40.9)	< .001
Comfortable talking to parent/caretaker about worries	2,854 (49.9)	205 (46.2)	181 (48.0)	336 (42.4)	348 (43.9)	606 (63.2)	656 (64.1)	221 (37.1)	301 (41.0)	< .001
Very close to parent/caretaker	3,446 (60.8)	317 (73.4)	255 (70.8)	425 (54.5)	460 (58.6)	597 (62.5)	635 (62.1)	327 (55.2)	430 (58.2)	< .001
Neighborhood and peer factors										
Neighbors look out for one another	2,677 (49.9)	107 (28.9)	94 (29.8)	380 (48.8)	373 (48.6)	480 (55.6)	556 (57.6)	326 (55.6)	361 (49.9)	< .001
Unsafe school/neighborhood	1,214 (21.4)	128 (28.9)	158 (42.8)	99 (12.4)	96 (12.2)	328 (34.5)	231 (22.6)	88 (15.1)	86 (11.8)	< .001
Adult help is available	2,157 (39.1)	252 (62.7)	220 (61.3)	394 (51.8)	404 (53.3)	260 (27.2)	221 (21.7)	170 (30.4)	236 (33.3)	< .001
Peers smoke or drink	1,235 (25.5)	104 (25.4)	78 (23.1)	130 (19.8)	148 (22.1)	132 (14.7)	92 (10.1)	320 (70.8)	231 (45.8)	< .001
Peer have started a fight	3,433 (67.3)	309 (81.7)	212 (74.4)	390 (56.9)	349 (50.7)	738 (77.3)	773 (75.8)	348 (67.8)	314 (54.6)	< .001

- indicates that variable was not assessed in site and/or survey round.

**Table 4:** Restricted logistic regression results by site and sex^a^

Factor	Pooled odds ratio (95% CI)	Flanders, Belgium		Shanghai, China		Kinshasa, DRC		Semarang, Indonesia	
		Male	Female	Male	Female	Male	Female	Male	Female
Associated with increased odds of violence perpetration									
Individual-level factors									
High media consumption	1.36∗∗∗ (1.18–1.56)	1.29 (0.84–2.00)	1.89 (0.93–3.84)	1.51 (0.89–2.58)	1.25 (0.62–2.51)	1.69∗∗∗ (1.25–2.27)	1.14 (0.87–1.51)	1.46∗ (1.01–2.10)	1.29 (0.84–1.98)
Viewed pornography	3.07∗∗∗ (2.41–3.89)	2.76∗∗∗ (1.70–4.48)	2.25 (0.98–5.14)	-	-	-	-	3.50∗∗∗ (2.39–5.14)	3.35∗∗∗ (2.08–5.41)
Victim of violence or bullying	7.03∗∗∗ (5.99–8.24)	3.91∗∗∗ (2.48–6.16)	3.72∗∗∗ (1.89–7.34)	8.76∗∗∗ (4.55–16.86)	9.04∗∗∗ (4.26–19.18)	9.38∗∗∗ (6.84–12.86)	8.38∗∗∗ (6.19–11.34)	6.12∗∗∗ (3.65–10.25)	6.28∗∗∗ (3.61–10.90)
Ever drank alcohol	3.27∗∗∗ (2.66–4.01)	2.79∗∗∗ (1.74–4.48)	1.60 (0.79–3.26)	2.96∗∗∗ (1.83–4.80)	3.01∗∗∗ (1.63–5.57)	3.64∗∗∗ (2.35–5.63)	4.85∗∗∗ (2.73–8.62)	13.33∗∗∗ (3.77–47.04)	20.00∗∗ (2.21–181.01)
Individual mental health and adverse experiences									
Depression: 2–3 symptoms	1.44∗∗∗ (1.24–1.67)	1.08 (0.63–1.85)	2.07∗ (1.02–4.20)	2.09∗∗ (1.22–3.56)	1.82 (0.80–4.13)	1.76∗∗∗ (1.30–2.38)	1.41∗ (1.04–1.92)	1.00 (0.67–1.50)	1.40 (0.89–2.20)
Depression: 4–6 symptoms	2.19∗∗∗ (1.76–2.72)	2.12 (0.90–5.01)	2.59∗ (1.06–6.32)	2.57∗∗ (1.37–4.83)	4.77∗∗∗ (2.17–10.52)	2.55∗∗ (1.41–4.59)	1.41 (0.69–2.91)	1.28 (0.76–2.15)	2.90∗∗∗ (1.73–4.87)
Adverse childhood experiences: 1 ACE	1.64∗∗∗ (1.27–2.11)	-	-	2.52 (0.86–7.34)	2.17 (0.19–24.16)	1.44 (0.94–2.19)	1.87∗∗ (1.24–2.82)	1.71 (0.72–4.06)	1.32 (0.46–3.84)
Adverse childhood experiences: 2–3 ACEs	2.44∗∗∗ (1.94–3.07)	-	-	2.35 (0.88–6.26)	8.06∗ (1.04–62.59)	2.11∗∗∗ (1.43–3.12)	2.37∗∗∗ (1.63–3.46)	3.87∗∗∗ (1.82–8.22)	3.11∗ (1.28–7.54)
Adverse childhood experiences: 4–5 ACEs	5.14∗∗∗ (3.97–6.68)	-	-	5.26∗∗ (1.92–14.37)	36.21∗∗∗ (4.79–273.51)	3.61∗∗∗ (2.25–5.79)	3.68∗∗∗ (2.24–6.03)	6.57∗∗∗ (3.01–14.36)	10.12∗∗∗ (4.08–25.08)
Adverse childhood experiences: 6 + ACEs	10.54∗∗∗ (7.45–14.91)	-	-	20.38∗∗∗ (6.58–63.10)	72.31∗∗∗ (8.36–625.76)	5.87∗∗∗ (2.90–11.86)	8.53∗∗∗ (4.29–16.96)	6.81∗∗∗ (2.80–16.54)	38.09∗∗∗ (12.41–116.87)
Agency/empowerment factors									
High behavioral control	1.49∗∗∗ (1.30–1.70)	1.17 (0.75–1.83)	1.03 (0.53–2.00)	1.02 (0.64–1.63)	1.38 (0.75–2.56)	2.08∗∗∗ (1.59–2.72)	1.82∗∗∗ (1.39–2.40)	1.11 (0.76–1.62)	1.33 (0.89–1.96)
High decision-making	1.34∗∗∗ (1.16–1.55)	0.86 (0.55–1.35)	0.92 (0.45–1.85)	2.20∗∗ (1.32–3.66)	1.04 (0.55–1.97)	1.36∗ (1.03–1.80)	1.45∗∗ (1.10–1.90)	1.48 (0.96–2.28)	1.18 (0.73–1.92)
Neighborhood and peer factors									
Unsafe school/neighborhood	1.99∗∗∗ (1.71–2.31)	1.86∗∗ (1.19–2.89)	1.47 (0.79–2.75)	5.15∗∗∗ (3.05–8.71)	2.88∗∗ (1.43–5.80)	2.05∗∗∗ (1.56–2.70)	1.94∗∗∗ (1.43–2.64)	1.94∗∗ (1.21–3.11)	1.40 (0.80–2.45)
Peers smoke or drink	2.05∗∗∗ (1.73–2.44)	4.14∗∗∗ (2.51–6.82)	1.17 (0.54–2.54)	2.64∗∗∗ (1.53–4.56)	6.15∗∗∗ (2.97–12.76)	1.71∗∗ (1.17–2.48)	1.31 (0.83–2.05)	1.86∗ (1.15–3.00)	1.90∗∗ (1.18–3.06)
Peers have started a fight	5.88∗∗∗ (4.75–7.28)	3.53∗∗∗ (1.69–7.40)	3.13∗ (1.07–9.17)	12.59∗∗∗ (5.02–31.56)	7.26∗∗∗ (2.81–18.77)	8.47∗∗∗ (5.22–13.74)	5.57∗∗∗ (3.54–8.77)	4.87∗∗∗ (2.88–8.24)	4.46∗∗∗ (2.69–7.39)
Associated with decreased odds of violence perpetration									
Individual-level factors									
Egalitarian views on sexual double standard	0.60∗∗∗ (0.50–0.73)	0.62 (0.35–1.10)	0.53 (0.20–1.42)	0.82 (0.45–1.47)	b	0.64∗ (0.45–0.91)	0.37∗∗∗ (0.20–0.66)	0.69 (0.44–1.08)	0.77 (0.46–1.30)
Egalitarian views on gender stereotypical traits	0.72∗∗ (0.59–0.88)	0.78 (0.38–1.59)	0.27∗ (0.09–0.79)	1.00 (0.46–2.17)	0.70 (0.27–1.81)	0.81 (0.55–1.19)	0.64 (0.39–1.04)	0.78 (0.47–1.30)	0.89 (0.52–1.51)
Agency/empowerment factors									
High youth voice	0.85∗ (0.75–0.98)	0.35∗∗∗ (0.21–0.58)	0.52∗ (0.27–0.97)	0.97 (0.60–1.58)	0.39∗ (0.19–0.80)	1.20 (0.93–1.56)	0.88 (0.67–1.16)	0.98 (0.67–1.44)	1.02 (0.69–1.51)
Family-level factors									
High parental monitoring	0.57∗∗∗ (0.50–0.66)	0.50∗∗ (0.31–0.82)	0.84 (0.45–1.57)	0.58∗ (0.34–0.96)	0.34∗∗ (0.18–0.66)	0.50∗∗∗ (0.38–0.66)	0.67∗∗ (0.51–0.87)	0.61∗ (0.40–0.93)	0.56∗∗ (0.37–0.85)
Comfortable talking to parent/caretaker about worries	0.62∗∗∗ (0.54–0.71)	0.55∗∗ (0.36–0.85)	0.62 (0.33–1.18)	0.35∗∗∗ (0.20–0.61)	0.39∗∗ (0.19–0.78)	0.72∗ (0.55–0.95)	0.67∗∗ (0.51–0.88)	0.81 (0.56–1.19)	0.48∗∗∗ (0.31–0.73)
Very close to parent/caretaker	0.59∗∗∗ (0.52–0.68)	0.55∗ (0.35–0.87)	0.63 (0.32–1.23)	0.61∗ (0.37–0.99)	0.34∗∗∗ (0.18–0.65)	0.81 (0.62–1.06)	0.49∗∗∗ (0.37–0.64)	0.72 (0.50–1.04)	0.46∗∗∗ (0.31–0.69)
Neighborhood/peer factors									
Neighbors look out for one another	0.78∗∗∗ (0.68–0.90)	0.78 (0.47–1.30)	0.71 (0.33–1.56)	0.98 (0.60–1.57)	0.33∗∗ (0.17–0.67)	1.02 (0.78–1.35)	0.81 (0.61–1.07)	0.66∗ (0.46–0.95)	0.60∗ (0.41–0.90)
Adult help is available	0.81∗∗ (0.70–0.94)	0.39∗∗∗ (0.25–0.62)	0.73 (0.39–1.37)	1.07 (0.67–1.72)	0.44∗ (0.22–0.85)	1.04 (0.77–1.39)	0.57∗∗ (0.40–0.81)	1.32 (0.89–1.96)	1.03 (0.68–1.56)
Mixed associations									
Family-level factors									
Married parents	0.99 (0.84–1.16)	0.66 (0.41–1.08)	1.24 (0.56–2.71)	0.62 (0.32–1.21)	0.36∗∗ (0.18–0.71)	1.31∗ (1.00–1.72)	1.10 (0.84–1.45)	1.39 (0.65–2.98)	0.37∗∗ (0.20–0.68)
Unique associations									
Individual-level factors									
High academic performance	0.97 (0.85–1.11)	0.63∗ (0.41–0.98)	1.05 (0.55–2.02)	0.96 (0.60–1.53)	0.64 (0.34–1.21)	1.12 (0.86–1.45)	0.96 (0.73–1.26)	0.87 (0.58–1.32)	1.36 (0.91–2.05)
Family-level factors									
At least one parent employed	0.96 (0.78–1.18)	0.41∗ (0.19–0.92)	3.56 (0.47–27.02)	0.83 (0.10–6.74)	b	0.88 (0.64–1.22)	1.07 (0.74–1.54)	1.26 (0.63–2.55)	0.84 (0.40–1.74)
No association									
Individual-level factors									
Egalitarian views on gender stereotypical roles	1.05 (0.83–1.31)	-	-	0.66 (0.23–1.88)	1.08 (0.55–2.10)	1.10 (0.74–1.64)	0.96 (0.63–1.47)	1.43 (0.71–2.87)	1.21 (0.59–2.48)
Family-level factors									
High parental education	1.07 (0.93–1.23)	1.35 (0.78–2.31)	0.66 (0.31–1.41)	0.83 (0.51–1.36)	0.87 (0.47–1.60)	1.16 (0.88–1.52)	1.03 (0.79–1.36)	1.29 (0.89–1.87)	0.95 (0.62–1.45)
High parental educational expectations	1.07 (0.86–1.33)	-	-	-	-	1.30 (0.74–2.27)	0.68 (0.42–1.09)	1.14 (0.79–1.65)	1.23 (0.82–1.84)

∗*p* < .05, ∗∗*p* < .01, ∗∗∗*p* < .001.

a Pooled estimates adjusted for site, sex, and age; site-specific and sex-specific estimates adjusted for age.

b No estimate could be generated because independent variable perfectly predicted failure.

**Table 5:** Full multivariable logistic regression (adjusted odds ratio) results by site^a^

Factor	Flanders, Belgium	Shanghai, China	Kinshasa, DRC	Semarang, Indonesia
Demographic characteristics				
Age in years	b	b	b	b
Female sex	0.50 (0.21–1.14)	0.45∗ (0.22–0.94)	1.02 (0.78–1.33)	0.95 (0.52–1.74)
Individual-level factors				
High media consumption	1.04 (0.50–2.17)	0.91 (0.43–1.96)	1.15 (0.87–1.51)	1.39 (0.74–2.61)
Viewed pornography	1.35 (0.62–2.96)	-	-	2.47∗∗ (1.31–4.67)
Egalitarian views on sexual double standard	0.78 (0.29–2.08)	1.14 (0.50–2.60)	0.60∗ (0.39–0.90)	0.72 (0.33–1.57)
Egalitarian views on gender stereotypical traits	0.65 (0.24–1.79)	0.94 (0.37–2.37)	0.89 (0.58–1.37)	1.78 (0.51–6.19)
Egalitarian views on gender stereotypical roles	-	1.49 (0.60–3.71)	1.05 (0.69–1.61)	1.53 (0.55–4.31)
Victim of violence or bullying	2.86∗∗ (1.32–6.17)	5.10∗∗∗ (2.43–10.71)	6.05∗∗∗ (4.55–8.04)	6.38∗ (1.05–38.90)
High academic performance	0.77 (0.40–1.52)	0.91 (0.52–1.60)	1.24 (0.94–1.65)	0.87 (0.41–1.85)
Ever drank alcohol	2.63∗ (1.07–6.42)	2.48∗ (1.22–5.01)	3.26∗∗∗ (1.97–5.39)	19.38∗ (1.74–215.96)
Individual mental health and experiences				
Depression: 2–3 symptoms	0.40 (0.11–1.42)	1.14 (0.58–2.25)	1.09 (0.81–1.47)	0.98 (0.47–2.04)
Depression: 4–6 symptoms	1.17 (0.36–3.79)	1.05 (0.49–2.26)	0.95 (0.51–1.77)	1.09 (0.49–2.40)
Adverse childhood experiences: 1 ACE	-	1.98 (0.48–8.18)	1.43 (0.97–2.10)	0.52 (0.13–2.00)
Adverse childhood experiences: 2–3 ACEs	-	1.33 (0.37–4.78)	1.51∗ (1.05–2.17)	0.60 (0.14–2.59)
Adverse childhood experiences: 4–5 ACEs	-	2.86 (0.78–10.46)	1.48 (0.93–2.34)	1.26 (0.33–4.83)
Adverse childhood experiences: 6 + ACEs	-	2.61 (0.52–13.11)	3.08∗∗ (1.49–6.36)	0.63 (0.10–3.91)
Agency/empowerment factors				
High youth voice	0.52 (0.25–1.09)	1.18 (0.60–2.30)	1.00 (0.77–1.31)	1.65 (0.74–3.68)
High behavioral control	1.41 (0.71–2.81)	1.41 (0.78–2.54)	1.39∗ (1.05–1.84)	0.80 (0.44–1.44)
High decision-making	0.71 (0.33–1.53)	1.41 (0.78–2.56)	1.16 (0.87–1.54)	0.66 (0.36–1.20)
Family/household-level factors				
Married parents	1.51 (0.68–3.34)	0.72 (0.34–1.51)	1.04 (0.80–1.35)	b
High parental education	1.25 (0.57–2.75)	0.89 (0.49–1.60)	1.07 (0.82–1.38)	1.04 (0.53–2.03)
High parental educational expectations	b	b	b	0.81 (0.37–1.76)
At least one parent employed	b	b	0.81 (0.58–1.13)	b
High parental monitoring	0.83 (0.39–1.80)	0.72 (0.35–1.44)	0.57∗∗∗ (0.44–0.74)	0.80 (0.40–1.61)
Comfortable talking to parent/caretaker about worries	0.66 (0.27–1.60)	0.51 (0.25–1.01)	1.04 (0.78–1.38)	0.60 (0.28–1.28)
Very close to parent/caretaker	0.92 (0.40–2.10)	0.88 (0.46–1.69)	0.79 (0.59–1.04)	0.74 (0.41–1.33)
Neighborhood and peer factors				
Neighbors look out for one another	0.88 (0.38–2.00)	1.27 (0.68–2.37)	0.95 (0.73–1.24)	0.67 (0.35–1.28)
Unsafe school/neighborhood	0.99 (0.45–2.20)	1.80 (0.95–3.40)	1.17 (0.88–1.56)	0.93 (0.40–2.17)
Adult help is available	1.34 (0.59–3.05)	1.14 (0.61–2.14)	0.76 (0.55–1.04)	1.39 (0.76–2.56)
Peers smoke or drink	1.86 (0.71–4.90)	1.27 (0.65–2.46)	0.99 (0.67–1.45)	2.01∗ (1.05–3.84)
Peers have started a fight	2.96∗ (1.02–8.54)	4.21∗∗∗ (1.79–9.91)	3.35∗∗∗ (2.25–4.97)	3.14∗∗ (1.45–6.80)

- indicates that variable was not assessed in site and/or survey round.

∗*p* < .05, ∗∗*p* < .01, ∗∗∗*p* < .001.

a Site-specific odds ratios and 95% confidence intervals; analyses adjusted for all variables with estimates presented in the site column. Sample sizes vary due to multiple imputations: site Ns are 231–511 (Flanders), 817–1,347 (Shanghai), 1,481–1,858 (Kinshasa), and 457–1,020 (Semarang).

b Variable was excluded from analysis due to VIF more than 10.

For these logistic regression analyses, all independent variables were dichotomized except the depression symptoms sand ACEs indices, which were partially collapsed and included as nominal categorical variables to allow for variation in the odds ratio magnitude across categories. Both restricted-model and full-model regressions used multiple imputations to minimize bias introduced by missing data. All analyses were conducted in Stata, version 16.1 [21].

After restricted-model odds ratios were estimated, we categorized the observed associations as follows:

• *Increased odds of violence perpetration*: Variables with a pooled odds ratio significantly greater than one, or multiple site-specific and sex-specific odds ratios significantly greater than one;
• *Decreased odds of violence perpetration*: Variables with a pooled odds ratio significantly less than one, or multiple site-specific and sex-specific odds ratios significantly less than one;
• *Mixed associations*: Variables with a nonsignificant pooled odds ratio, at least one site-specific and sex-specific odds ratio significantly greater than one, and at least one site-specific and sex-specific odds ratio significantly less than one;
• *Unique association*: Variables with only one significant site-specific and sex-specific odds ratio; and
• *No association*: Variables without significant odds ratios.

## Results

### Descriptive findings

The sample was evenly split by sex, with 2,823 boys (49.0%) and 2,939 girls (51.0%). Adolescents were 12.3 years old on average, with a range from 10 to 14 years. As noted in Table 2, violence perpetration varied significantly by site, sex, and age. Overall, one-fourth of boys (25.9%) reported violence perpetration in the past six months, compared with about one in six girls (17.6%). Rates were highest among 10-year-old adolescents (31.4%), followed by 14-year-olds (23.2%). The site with the greatest level of reported violence perpetration was Kinshasa (33.4%) and Shanghai had the lowest level (7.8%).

Table 3 presents the distribution of the key independent variables by sex and geography. Chi-squared tests of association showed there were significant variations by site and/or sex for every variable, although the extent of the variation was not consistent.

### Restricted-model findings

Eleven factors were found to be associated with *increased violence perpetration* in multiple sites: high media consumption, having viewed pornography, being a victim of violence or bullying, having ever drank alcohol, reporting any depressive symptoms, having any ACEs, greater behavioral control, greater decision-making, feeling unsafe in the neighborhood/school, having peers who smoke or drink, and witnessing peers start a fight.

Conversely, eight factors were found to be associated with *decreased violence perpetration*: more egalitarian views on the sexual double standard and gender stereotypical traits scales; greater youth voice; greater levels of parental monitoring, closeness, and comfort discussing worries; having neighbors who look out for one another, and greater perceived availability of adult help.

One factor was found to have *mixed associations* with violence perpetration: having married parents was associated with decreased odds of violence perpetration for girls in Shanghai and Semarang but increased odds for boys in Kinshasa. In addition, two factors–high academic performance and having at least one parent who works for pay had a *unique* association specific to one site or sex subgroup, boys in Flanders. Specifically, these factors were associated with decreased odds of violence among boys in Flanders. No significant associations were found for holding egalitarian views on gender stereotypical roles, high parental education, and high parental educational expectations for the adolescent.

There was a strong and dose-related relationship in the odds of violence perpetration, with increasing number of ACEs for both boys and girls, where data were available (all sites but Flanders). Across these sites, about 15% of adolescents reported 4–5 adverse experiences and had roughly five times the odds of violence perpetration compared with those who reported no ACEs, an association that was particularly pronounced for girls.

Across the variables explored, there was a substantial variation in the observed associations by site and sex. In addition to ACEs, the only other variables with a consistent association for all sites and sex groups were being a victim of violence or bullying and witnessing peers start a fight, which had significant positive associations with violence perpetration regardless of a subgroup.

### Full-model findings

**Figure 1:**
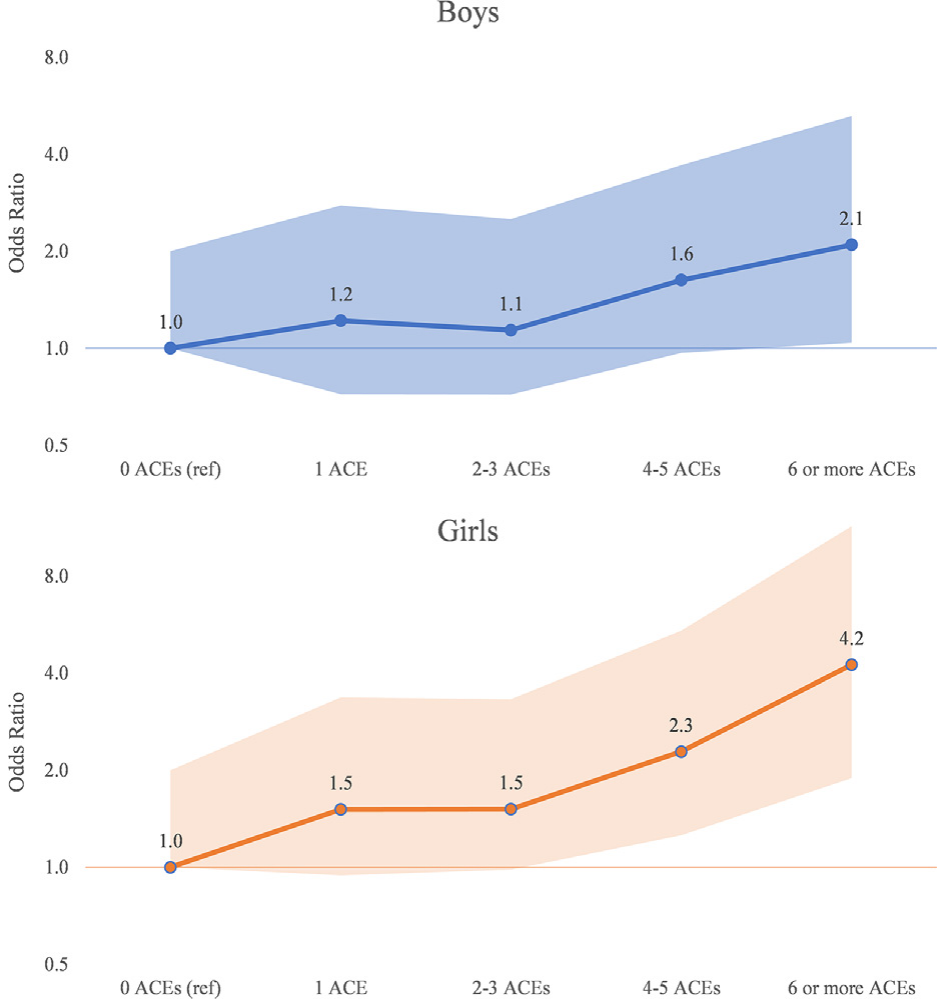
Adjusted relative odds of violence perpetration, by sex and number of reported ACEs.

Table 5 presents the results of the site-specific multivariable regressions. Age was excluded from all models due to a high VIF in each site. Across all four sites, three factors retained significant positive associations with violence perpetration: being a victim of violence or bullying, having ever drank alcohol, and witnessing peers start a fight. Other factors—including experiencing an increased number of ACEs, holding egalitarian views on the sexual double standard, high parental monitoring, and having peers who smoke or drink—had significant associations in one of the site models. Cross-site associations between violence perpetration and ACEs are presented for boys and girls in Figure 1.

## Discussion

Our findings indicate a wide variation in peer violence perpetration by young adolescents across low-income, middle-income, and high-income countries. Perpetration in the past six months ranged from 7.8% in Shanghai to 33.4% in Kinshasa; variations that may indicate correspondence between peer and partner violence perpetration at the regional level. In a study of four cities worldwide, Peitzmeier et al. [22] reported prevalence of partner violence in adolescents aged 15–19 years ranging from 9% in Shanghai to roughly 40% in Johannesburg. Despite the differences in the specific violence outcome and study design, this variation appears similar to the regional differences observed in the present study. What distinguishes the present findings from those of others is that the literature largely focuses on male-on-female violence [23]. Growing United States–based evidence suggests violence occurs both within and across genders [24], although the present study does not identify the gender of the victim.

Much of the research on youth violence perpetration has focused on psychological and emotional correlates. A meta-analysis on youth violence in low-income and moderate-income countries found victimization was one of the factors associated with violence involvement [12]. Consistent with that research, we find a cross-cutting relationship between experiencing violence and perpetrating it. This inter-relationship was also found by Ramaiya et al. [25] in both the DRC and Malawi, although those results indicated a greater association for boys than for girls. In the present study, the inter-relationship between perpetrating violence when having been victimized was consistent and considerable, with subgroup odds ratios of at least 3.7. This study builds on prior research of this relationship [12,26], by identifying it in girls and boys, across a multicountry sample.

Zimmerman et al. [18] found that in Kinshasa, DRC measures of agency were not consistently associated with prosocial acts; this study indicates greater behavioral control and decision-making may be associated with violence perpetration for multiple subgroups. It is plausible that under certain circumstances, empowered young people may use their power to perpetrate violence. In addition, it may be that in certain contexts violence perpetration may be protective against unwanted aggression. The associations found for our measures of agency underscore the need for further research into these multifaceted constructs.

The association between ACEs and violence perpetration is extremely pronounced in our analyses. Independent of geography or sex of respondent, as the number of ACEs increases, so too do the odds of perpetrating violence. While the magnitude of this association varied by site, it was present across all those with ACEs data (all but Flanders). A previous GEAS study reported the high prevalence of ACEs in pilot samples of early adolescents [15], which is reflected in our sample as well. In each site with ACEs data, at least 14% of respondents reported at least four adverse experiences. Another recent study of three Indonesian communities found a significant association between ACEs and violence perpetration [16].

Despite vast geographic, economic, and linguistic variations, in the present analyses there was a direct relationship between the number of adverse exposures as a child and violence perpetration as a young adolescent in all sites. Fagan reported that those who experienced a greater number of ACEs before the age of 12 years had 50% greater odds of experiencing violence victimization as older adolescents; while this outcome is victimization rather than perpetration, the associations parallel ours [27]. The work of Kidman and Kohler in Malawi has shown the strong association between cumulative childhood adversity and intimate partner violence for both boys and girls [28]. Together, these findings indicate that the accumulation of ACEs may increase the odds of violence victimization and perpetration, creating a double disadvantage for young people experiencing such trauma.

Another cross-cutting finding of the present analyses is the relationship between pornography viewing and violence perpetration for both boys and girls. This finding held true for boys and girls in Semarang, Indonesia and boys in our high-income country sample (Flanders, Belgium). Current research examining the relationship between pornography and violence tends to focus on sexual or dating violence and is largely based in North America [29]. In the present analyses, odds ratios associated with having viewed pornography ranged from 2.8 for boys in Flanders to 3.5 for boys in Semarang. Given the age group of our sample, it is possible that this is a marker of social deviance; however, given the prevalence, may be other dynamics at play. Without knowing the content of what is being viewed, it is not possible to draw definitive conclusions.

The present analyses also identified factors that were commonly associated with significantly lower odds of violence perpetration. Specifically, several parent factors were significantly associated with diminished peer violence perpetration. These findings reinforce those of Kaufman-Parks et al. [30] and a review by Lösel and Farrington [31], which found parent relationships are likely to be protective against youth violence. It is possible other parental factors—such as their own attitudes toward violence or egalitarian gender views—may be related to adolescent violence perpetration. While such data were not available for the present study, they may be worthwhile avenues for future research.

Likewise, endorsement of more egalitarian views on two gender norms scales was significantly associated with less reported violence perpetration. Similar associations have been found in United States–based studies of relational violence and aggression [32,33]. Based on those and similar data, Miller has concluded that addressing unequal gender norms may be a powerful way to impact sexual violence [34]. As Ramaiya et al. [16] have noted in Indonesia, this may be especially true for boys. With limited international data to date, it is difficult to draw a similar conclusion globally.

### Limitations

The analyses presented in this study are based on cross-sectional observational data and, as such, should be interpreted as descriptive risk and protective factors. The restricted-model analyses reported in Table 4 are likely subject to confounding, given that controls and/or stratification were only introduced for age, sex, and site. While the full models reported in Table 5 provide an insight into associations of the other factors considered in this study, there is still a possibility of residual confounding. Although several of the independent variables had missing responses exceeding 3%, the use of multiple imputations in our regression models minimizes bias resulting from these missing data.

While this study incorporated numerous factors at multiple ecological levels, the variables included are not exhaustive. Factors not included here, such as parent attitudes toward violence and the content of pornography viewed, may be meaningful and could be examined in future research.

Some aspects of the study sample may also be considered limitations. Due to variation in the site-specific sample size, some sites may lack statistical power to detect associations with the independent variables of interest. Violence perpetration was especially rare among girls in Shanghai; thus, estimates could not be generated for several variables among that subgroup. Finally, the sample is not intended to be representative of young adolescents at the national or even the wider community level; rather, the samples were drawn from urban young people living in the most economically impoverished sections of their cities. The school-based sampling strategy used in this study does not specifically recruit adolescents involved in child welfare or judicial systems and does not capture adolescents who do not attend school; these adolescents may be at a higher risk of other negative outcomes than their peers. Future research that draws on samples of adolescents that are longitudinal or more representative, or that work to identify causal mechanisms, would help contextualize the findings of the present study.

### Conclusion

Using this large, multicountry survey of young adolescents in low-income communities, we sought to identify common and unique factors related to violence perpetration. What we see are striking commonalities across these diverse cultures and geographies. It is evident from the present research that those who grow up in family, school, and neighborhood environments where they feel vulnerable are more likely to perpetuate violence. Specifically, everywhere we look those exposed to more adversity as children are more likely to perpetuate violence as young adolescents. Furthermore, pornography viewing in this age group is associated with violence perpetration, as is alcohol consumption. Whether these are “transitional behaviors,” as Jessor referred to them [35], or markers of greater social deviance awaits further research. Based on the current data, we also know that those who hold more egalitarian views report less violence perpetration. This exploratory study identifies numerous opportunities for further research, including how these factors associate with violence over time and how interventions may leverage them to prevent adolescent violence.
